# Herpes Virus, Oral Clinical Signs and QoL: Systematic Review of Recent Data

**DOI:** 10.3390/v11050463

**Published:** 2019-05-21

**Authors:** Salvatore Crimi, Luca Fiorillo, Alberto Bianchi, Cesare D’Amico, Giulia Amoroso, Francesca Gorassini, Roberta Mastroieni, Stefania Marino, Cristina Scoglio, Francesco Catalano, Paola Campagna, Salvatore Bocchieri, Rosa De Stefano, Maria Teresa Fiorillo, Marco Cicciù

**Affiliations:** 1 Department of General Surgery and Medical Surgery Specialties University of Catania, 95100 Catania, Italy; torecrimi@gmail.com (S.C.); alberto.bianchi@unict.it (A.B.); paolacampagna91@gmail.com (P.C.); 2 Department of Biomedical and Dental Sciences, Morphological and Functional Images, School of Dentistry, University of Messina, Policlinico G. Martino, Via Consolare Valeria, 98100 Messina, Italy; lfiorillo@unime.it (L.F.); cesaredamico89@gmail.com (C.D.); amoroso.giulia@hotmail.it (G.A.); gorassinifrancesca@gmail.com (F.G.); robertamastroieni@gmail.com (R.M.); stefaniamarino50@gmail.com (S.M.); cristina.scoglio@gmail.com (C.S.); fracat98@gmail.com (F.C.); salvo.bocchieri@gmail.com (S.B.); 3 Multidisciplinary Department of Medical-Surgical and Odontostomatological Specialties, University of Campania “Luigi Vanvitelli”, 80121 Naples, Italy; 4 Department of Biomedical and Dental Sciences, Morphological and Functional Images, University of Messina, Policlinico G. Martino, Via Consolare Valeria, 98100 Messina, Italy; rsdestefano@libero.it; 5 Unit of Microbiology and Virology, North Health Center ASP 5, 89100 Reggio Calabria, Italy; mariatfiorillo@hotmail.com

**Keywords:** Herpesviridae, Herpes labialis, oral health, Quality of Life, Viruses, HSV-1, HSV-2

## Abstract

This manuscript aims to highlight all the clinical features of the herpes virus, with a particular focus on oral manifestations and in the maxillofacial district about Herpes Simplex Virus-1 (HSV-1) and Herpes Simplex Virus-2 (HSV-2). Oral herpes virus is a very common and often debilitating infectious disease for patients, affecting oral health and having important psychological implications. The collection of relevant data comes from the scientific databases Pubmed, Embase; initially this collection obtained an extremely high number of results, 1415. After applying the inclusion and exclusion criteria, as well as a manual screening, the results included in this review were limited to 14. The results were expressed by evaluating all the signs and symptoms that this pathology entails during the study, paying attention to the characteristics linked to the quality of life and the psychological implications. This pathology has numerous therapies, which often make the healing phase of the manifestations of this viral pathology more comfortable. The therapies currently used for the treatment of this viral infection are pharmacological, topical, systemic, or instrumental, for example with laser devices.

## 1. Introduction

### 1.1. Background

Herpes viruses or herpesviruses are double-stranded DNA viruses with icosahedral symmetry, belonging to the Herpesviridae family. The main characteristic of this family of viruses is to go against replication within the host cells, without ever being completely eliminated, thus causing a latent infection. This clinical event occurs in a time that varies according to the type of the virus and to the sensitivity of the host. The virus can be reactivated from this latent state, even after many years, giving rise to a recurrence of the disease with a clinical manifestations. The stimuli that induce the “awakening” of viral activity can be heat, cold, trauma, fever, stress [1] and above all changes in a host’s immune defense status. In the virions, 120–200 nm in diameter, the core is distinguished, containing the DNA, the icosahedral symmetry capsid, the protein integument, and the envelope. The herpes simplex virus (HSV) is a species of virus, belonging to the Herpesviridae family, subfamily Alphaherpesvirinae, genus *Simplexvirus*. The first form, considerably diffused, is responsible for the appearance of characteristic febrile vesicles which normally affect the facial skin (lips, nostrils); it is also called herpes simplex labial. Initially, the cold sores manifest themselves with a slight tingling and a sense of heat on a reddened point of the lip. Within a few hours, some blisters filled with clear liquid, often painful, begin to develop in the same area as the initial point: The appearance of cold sores is ascertained [2,3,4]. The second form is a genital infection, also known as herpes genitalis. Both are contracted through physical and sexual contact. Given the location of virions in nerve ganglia, where they can remain quiescent for a long time, herpetic infection has recurrent characteristics in correspondence with stressful events of the immune system and usually reappears in the primary site [5,6,7,8].

### 1.2. Aim

The purpose of this manuscript is to evaluate the recent scientific literature concerning this specific field of oral pathology, that is, infections and pathologies related to the herpes virus, with focus on herpes virus 1 and 2 (HSV-1, HSV-2) [9]. The goal is to carry out a literature review by evaluating all the signs and symptoms related to this disease, so as to provide the clinician with a useful tool for an early and faster diagnosis of the disease and above all an appropriate therapeutic strategy, especially in case of correlations with other systemic pathologies [10,11,12]. In addition to the evaluation of the oral pathology proper, we will evaluate how this can affect the quality of life of the involved patients and above all how it can influence the psychological component of the latter as in other pathologies [13,14].

## 2. Materials and Methods

### 2.1. Application Protocol and Website Recording Data

A protocol including the investigation methods and the inclusion criteria for the current revision was submitted in the PROSPERO website, an international prospective register of systematic reviews. The parameters and the analytic structure of the present work can be visualized relating the CRD id and code; this systematic review has been submitted on the PROSPERO website platform. PROSPERO acknowledgement of receipt 133925 edited on 30/04/2019.

The data of this systematic investigation observed the Preferred Reporting Items for Systematic Review accordingly with the (PRISMA) statement.

### 2.2. Target Questions

The questions processed the following guidelines, according to PICO (Patient, Intervention, Comparison, Outcome):

- What is the oral manifestation of a herpes virus infection, and what about new therapies?
- Is there an implication in Quality of Life (QoL) and psychological profile?

### 2.3. Search Strategy

The literature investigation was performed in five different electronic databases, including Ovid MEDLINE, PubMed, and EMBASE. A manual search on Dentistry and Pharmacological sources was also conducted for relevant studies published.

Digital and searches by hand were performed on herpes virus and oral manifestation, with a secondary outcome in QoL and psychological implications. In-depth research of the reference lists in the recorded manuscripts was performed in order to add significant studies and to increase the sensitivity of the revision.

### 2.4. Collection Data

The Medical Subject Headings (MeSH) was applied for finding the keywords used in the present revision. The selected key words: (“Herpes virus” OR “HSV”) AND (“oral” OR “QoL”) was recorded for collecting the data. The date of last search with these results is 30 April 2019.

### 2.5. Manuscript Selections

Two independent reviewers of two different universities (Messina and Catania) singularly analyzed the obtained papers in order to select inclusion and exclusion criteria as follows: Reviewers correlated their evaluations and analyzed differences through comparing the manuscripts and consulting a third experienced senior independent reviewer (M.C., University of Messina) when consensus could not be reached. For the stage of the full-text articles revision, a complete independent dual analysis was performed.

### 2.6. Research Classifications

The method of classification included all human prospective and retrospective clinical studies, split mouth cohort studies, case-control papers, and case series manuscripts, animal investigations and literature review published between February 2009 and April 2019 on herpes virus infection clinical signs and systemic implications.

### 2.7. Exclusion and Inclusion Criteria

The full texts of all studies related to the main revision topics were obtained comparing the inclusion parameters:

- Herpes virus infection oral manifestations and therapies;
- QoL and psychological implications of this infection;
- Herpes virus clinical signs and therapy.

The following exclusion criteria were applied:

- Patients with other specific disease as osteoporosis, immunologic disorders, uncontrolled diabetes mellitus, or other surgical risk related systemic conditions;
- Not enough information regarding the topic;
- Animal or in vitro studies;
- Articles published prior to 1 February 2009;
- No access to the title and abstract.

### 2.8. Strategy for Collecting Data

After the first literature analysis, the entire manuscript titles list was highlighted to exclude irrelevant publications, case reports and non-English language publications. Then, research was excluded based on data obtained from screening just the abstracts. The final selection was performed reading the full texts of the papers in order to approve each study’s eligibility, based on the inclusion and exclusion criteria.

### 2.9. Record of the Extracted and Collected Data Extraction

The results and conclusions of the selected full text papers were used for assembling the data, according to the aims and themes of the present revision, as listed onwards.

The following parameters were used as a method for assembling the data and then organized following the schemes as seen in Table 2:

- Author (Year)—authors and year;
- Sample Size—sample size number;
- Sample Groups—type of groups;
- Posology—therapy posology;
- Bias risk—type of study and induced risk of bias;
- Statistical results—statistical results.

### 2.10. Risk of Bias Assessment

The grade of bias risk was independently considered, as reported in literature [15,16,17,18].

Potential causes of bias were investigated:

- Selection bias;
- Performance bias and detection bias;
- Attrition bias;
- Reporting bias;
- Examiner blinding, examiner calibration, standardized follow-up description, standardized residual graft measurement, standardized radiographic assessment.

### 2.11. Herpes Virus Oral and Systemical Implications

#### 2.11.1. Disease Definition

Herpes simplex is a viral disease caused by the herpes simplex virus. Infections are classified accordingly to the part of the body affected: Cold sores can cause small blisters placed in groups and sore throats, whereas genital herpes presents with minimal symptoms such as small blisters that break and cause mild ulcers. The presentation of herpes symptoms occurs cyclically, with periods of active illness followed by asymptomatic periods. The first episode is often more severe and may be associated with fever, muscle pain, swollen lymph nodes and headaches. Over time the episodes of illness decrease both in frequency and in severity. Other disorders caused by herpes simplex include systemic diseases, just as it happens in other pathologies with oral and systemic correlations [13,14,19,20], among others, the herpetic paterecleris and herpetic encephalitis (Table 1).

There are two types of herpes simplex virus, type 1 (HSV-1) and type 2 (HSV-2). HSV-1 most commonly causes oral infections while HSV-2 most commonly affects the genitals. They are transmitted by direct contact with body fluids or through injuries to an infected individual. Transmission may also occur when symptoms are not present. Genital herpes is classified as a sexually transmitted disease. During delivery, herpes simplex can be transmitted to the baby. After infection, the viruses are transported along the sensory nerves to the bodies of nerve cells. The causes of recurrence can include decreased immune function, stress and exposure to the sun. Cold and genital herpes are usually diagnosed based on the presentation of symptoms. Diagnosis can be confirmed by viral culture or detection of herpes DNA in urine. Blood tests in search of antibodies against the virus may confirm a previous infection. The most effective way to avoid genital infections is to avoid vaginal, oral and anal sex. The use of condoms slightly decreases the risk. The daily intake of an antiviral drug by the infected person may reduce the spread. There is no vaccine available and, once infected, there is no cure. Paracetamol and topical lidocaine can be used to relieve symptoms. Treatments with antiviral drugs such as acyclovir or valaciclovir can reduce the severity of symptomatic episodes. Globally, rates among HSV-1 or HSV-2 adults are between 60% and 95%. HSV-1 is usually acquired during childhood. The incidence of HSV-1 is between 70% and 80% in populations of low socio-economic status and between 40% and 60% in those with a higher status. HSV infections cause several distinct medical disorders. Common infections of the skin or mucous membranes can affect the face and mouth (orofacial herpes), genital organs (genital herpes) or on the hands (herpetic fever). More serious disorders occur when the virus infects the eye (herpes keratitis) or invades the central nervous system, damaging the brain (herpetic encephalitis). People with immature or suppressed immune systems, such as newborns, transplant recipients or people with AIDS, are prone to get serious complications from HSV infections. These infections are also associated with cognitive deficits in bipolar disorder and Alzheimer’s disease, although this often depends on the genetics of the infected person. The spread of pathogens from the oral environment to the brain has recently been reported [21]. In all cases, HSV is never removed from the body by the immune system.

#### 2.11.2. Disease Clinics

Following a primary infection, the virus enters the nerves at the site of primary infection, moves towards the cellular body of the neuron and becomes latent in the ganglion. As a consequence of the primary infection, the body produces antibodies for the particular type of HSV involved, preventing a subsequent infection of this type in a different site. In individuals infected with HSV-1, seroconversion after an oral infection prevents further HSV-1 infections, such as the genital herpes, or herpes of the eye. HSV-1 seroconversion also appears to reduce the symptoms of subsequent HSV-2 infection, although HSV-2 may still be contracted. Many people infected with HSV-2 show no physical symptoms; these are called “asymptomatic”, or suffering from subclinical herpes. Herpes labialis is easily identified by a simple clinical examination of individuals with no previous history of lesions and who have had contact with an individual known to be HSV-1 positive. In these subjects, in general the vesicles are multiple, of round and superficial aspect, accompanied by an acute gingivitis. Adults with atypical presentation are more difficult to diagnose. The prodromal symptoms that occur before the appearance of herpetic lesions help to differentiate the symptoms of HSV from similar symptoms of other disorders, such as allergic stomatitis. When the lesions do not appear inside the mouth, primary oral-gold herpes are sometimes mistaken for impetigo, a bacterial infection. Even common mouth ulcers (aphthous ulcers) resemble intraoral herpes but do not present a vesicular phase.

#### 2.11.3. Diagnosis

Genital herpes can be more difficult to diagnose than oral herpes, since most people infected with HSV-2 do not have the classic symptoms. Furthermore, the diagnosis can be confused with many other conditions similar to genital herpes, including fungal infection, lichen planus, atopic dermatitis, and urethritis. Laboratory tests are often used to confirm a diagnosis of genital herpes. Laboratory tests include virus culture, immunofluorescence, skin biopsy and polymerase chain reaction to verify the presence of viral DNA. Although these procedures lead to highly sensitive and specific diagnoses, their high costs and time constraints discourage their common use in clinical practice. Until 1980, serological tests for HSV antibodies were rarely useful for diagnosis and were not routinely used in clinical practice. The old IgM serological tests were unable to distinguish between antibodies generated in response to HSV-1 or HSV-2 infections. However, a G-specific glycoprotein (IgG) test for HSV, was introduced in 1980 and has been shown to discriminate between HSV-1 from HSV-2 with a reliability of 98%. Herpes labialis or genitals should not be confused with further pathologies caused by other viruses of the Herpesviridae family, such as herpes zoster, which is due to the varicella-zoster virus. Differential diagnosis involves hand-foot-and-mouth disease [22]. The first phase corresponds to the perception of the warnings or the tingling and tingling. Furthermore, after careful observation of the lips, the skin appears already very tense and dry. After about a day, small vesicles begin to form which can then come together in a single larger bubble, causing the area to appear erythematous. On the third day it is possible to note an eruption of the vesicles, never to pierce the leakage of liquid and danger of serious infections. Finally, in the following days a crust is formed which will fall off on its own without leaving scars except, in some cases, a dark red patch. When the crust forms, unless it is touched, one is no longer infectious.

### 2.12. Herpes Virus Adopted Therapy

There is no method to eliminate the herpes virus from the body, but taking antiviral drugs can reduce the frequency, duration, and severity of outbreaks. Analgesics, such as ibuprofen and paracetamol (acetaminophen), can reduce pain and fever. Topical anesthetic treatments, such as prilocaine, lidocaine, benzocaine, or tetracaine, are also able to relieve itching and pain. Several antiviral drugs are effective for the treatment of herpes, including aciclovir, valaciclovir, famciclovir, and penciclovir. Aciclovir was the first to be discovered and is now available as an equivalent drug, as is valaciclovir. The evidence supports the use of aciclovir and valaciclovir for the treatment of cold sores, as well as for herpes infections in people with cancer. The evidence supporting the use of aciclovir in primary herpetic gingivostomatitis is weak. A number of topical antiviral drugs are effective for cold sores, including acyclovir, penciclovir, and docosanol. Lysine has been proposed for treatment based on good in vitro results; however, it has proved ineffective in humans. The cold sore virus, like other viruses encapsulated in a lipid membrane, turns out to be deactivated by long-chain saturated fatty alcohols. Of these, only docosanol has been approved by the Food and Drug Administration in the treatment of cold sores of the immunocompetent adult, having proved to be as effective as topical antivirals. Given the mechanism of action, it presents a risk of zero drug resistance minimum. The use of a trans-dermal electrical stimulator (TENS) on lesion sites has been suggested for the treatment of cold sores both in association with antiviral drugs and as a replacement [23,24]. Although no human data are available, authors would mention also nutraceutical treatment as novel strategy for HSV infections [25]. Plant-derived bioactive compounds, and more specifically polyphenols, have been demonstrated to exert marked anti-HSV activity and, among these, resveratrol would be considered a good candidate.

## 3. Results

The results obtained from a careful analysis of the literature, after applying the correct inclusion and exclusion criteria, were further evaluated by the authors. An independent analysis of the authors was carried out and the results compared further to evaluate their suitability. The initial research led to a high number of articles, 1415. Subsequently, applying the exclusion criteria, articles older than 10 years were removed first. At this point the remaining 261 articles were further skimmed by evaluating only the studies on humans (209), and subsequently only those available in full text format (63), so as to be able to consult all the available results and statistics. Only randomized clinical trials (RCTs) were included. At the end of the research 16 articles were obtained. Of these, only 14 papers contained sufficient information on the oral health of the patients considered, proposed innovative therapies, and spoke of herpes virus infections. The results obtained from the single articles have been summarized in Table 2, in such a way as to make the identification of efficient therapies or not quicker.

## 4. Discussion

### 4.1. Literature Review

According to Polansky et al. [26] the herbal treatment Gene-Eden-VIR/Novirin reduced the number and duration of outbreaks in oral herpes without adverse effect compared to acyclovir, valacyclovir and famciclovir drugs. It may be considered a good alternative according to authors. Semprini et al. [27] proposed a protocol to compare the use of kanuka honey to 5% acyclovir cream in the treatment of recurrent herpes simplex labialis. Palli et al. [28] in their 2017 study evaluated the use of squaric acid dibutyl ester (SADBE) immunotherapy for recurrent herpes labialis. The use of SADBE 2% was demonstrated to prevent herpes simplex virus outbreaks, according to them. Batavia et al. [29] evaluated the incidence of oral lesions and oral mucosa alterations on a HIV patients’ population. The early anti-retroviral therapy (ART) group obtained a lower incidence of oral lesion than the delayed ART group. They considered all types of oral lesions, not only HSV lesions. Batavia et al. considered viral infections lesions, idiopathic lesions and neoplasms as shown in the Table 2. According to Zhao et al. [30], topical formulation containing lipophilic catechins effectively inhibited herpes simplex labialis infection with clinical significance. During this trial, patients QoL showed significant differences between the placebo and AverTeaX formula groups, but significant differences were not found for pain, burning, bleeding or stress. You et al. [31] evaluated the effect of inosine pranobex versus acyclovir; the recurrence time of herpes labialis manifestation was lower in the inosine pranobex group but the total symptom score did not differ between the groups. Authors underlined that hyperuricemia was higher in the inosine pranobex group too. Miller and Westgate [32] conducted a medical laboratory screening collecting serum, urine and medical histories. They indicated that abnormal laboratory test results are common in dental office patients. They enrolled only patients that had a history of recurrent herpes labialis. Some dental procedures such as routine and invasive periodontal procedures might induce herpes labialis according to authors. The initial placement of orthodontic bands, brackets or wires, restorative procedures or tooth extraction, implant placement, biopsy, or bony resection could lead to an outbreak of herpes labialis. Bieber et al. [38] evaluated efficacy of acyclovir mucoadhesive tablet to labial herpes. With this therapy primary vescicular lesion was reduced and blocked herpes episodes were increased. Dougal et al. [39] evaluated the efficacy of low level light therapy with a 1072 nm infrared light for the treatment of herpes simplex labialis. Healing time with the use of laser was significant lower, with no difference between groups for time to lesion crusting. Low-level light therapy for oral mucosa pathology is also reported in literature [40]. According to Senti et al. [33] the topical application of an antiviral compound like hydroxypropyl-β-cyclodextrin did not reduce Herpes Labialis (HL) relapses. Khemis et al. [34] showed how CS20 had a superior effectiveness against functional symptoms associated to HSV-1 labial recurrences. The CS20 protective barrier gel improved the healing phases compared to topical acyclovir. They performed a visual analogue scale (VAS) and a pain intensity score highlighting how the placebo group had better results than the CS20 group in the first two days. Skulason et al. [35] studied the clinical activity of a gel combining monocaprin and doxycycline for HL treatment. According to them, the combination of monocaprin and low dose doxycycline offers an effective treatment for herpes labialis in both healing time and pain reduction. According to Munoz Sanchez et al. [36], 670nm low level laser therapy seemed to be an effective treatment without side effect for herpes labialis. Unfortunately, they did not perform a statistical analysis because of the large sample size, according to what they wrote. Busch et al. [37] evaluated the therapeutic effect of 1,5-pentanediol (PD) for herpes simplex labialis in a randomized, double blind clinical trial. PD gel did not show any prophylactic effect against herpes recurrence, but it demonstrated an effect against swelling and pain with no side effects.

### 4.2. Additional Information

All the international literature about this infectious disease has been screened following the inclusion criteria. The main causes of pain, burning and swelling which are incapacitating, with consequences at the systemic level and in the quality of life of the patients have been also recorded. Surely this pathology makes some dental practices difficult. Therefore, even simple maneuvers such as taking a dental impression are not feasible in the case of herpes simplex labialis or mucous in the active phase [41,42]. The surgery maneuvers may have an incidence in the exacerbation of the pathology [19,43,44,45,46,47,48,49,50,51,52], which could also have an influence linked to debilitating drug therapies for the patient or even to the patient’s own stress. Another important factor not reported in the literature and which could represent a study in the future is related to an evaluation of oral, oncological markers [53] or a simple evaluation of salivary pro-inflammatory cytokines [54] in the presence of this pathology and during the various stages of the pathology itself, with particular attention on the influence it can have on the oral bacterial flora [55] or on related pathologies related [56,57].

## 5. Conclusions

From the study conducted it is easy to see what the most efficient therapeutic strategies for this infectious disease are. Unfortunately, in the studies taken into consideration, few assessed the efficacy of the same drug, and therefore it was not possible to perform a statistical analysis. The most effective methods involve systemic or topical therapies in agreement with the authors. Also, the low-level laser therapy is not to be underestimated, as it can improve both healing time and all factors related to the quality of life of patients.

## Figures and Tables

**Table 1:** Herpes oral and systemic manifestations.

Pathology	Description	Example
Herpertic gingivostomatitis	Herpetic gingivostomatitis is often the initial presentation that occurs during the first herpes infection.	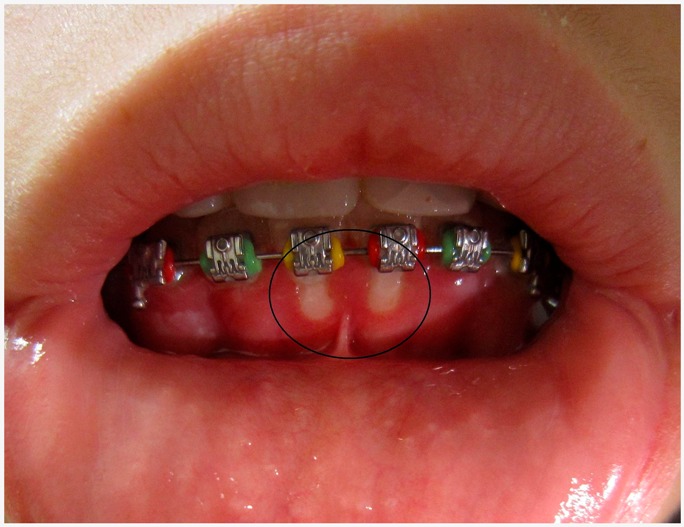 1. Herpetic gingivostomatitis CC BY-SA 3.0 licence, adapted, for concession of James Heilman, MD.
Herpes labialis	Infection occurs when the virus comes into contact with the oral mucosa or abraded skin.	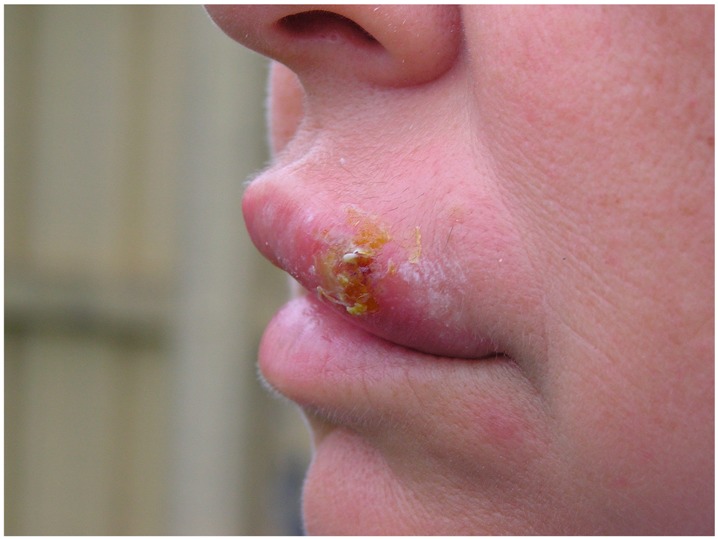 2. Herpes labialis manifestation, public domain photo, adapted for gentle concession of Ben Tillman.
Herpes genitalis	When symptomatic, the typical manifestation of HSV-1 or HSV-2 genital infection is characterized by clusters of papules and inflamed vesicles on the outer surface of the genitals that resemble those found in cold sores.	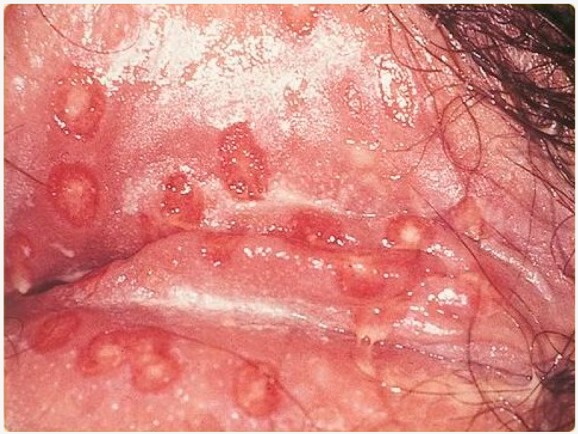 3. Herpes genitalis manifestations, licence CC BY-SA 3.0, adapted for gentle concession of SOA-AIDS Amsterdam.
Herpetic paterecleris	The herpetic paterecleris is a painful infection that usually affects the fingers or thumbs. Occasionally, infection occurs on the fingers. People who engage in contact sports such as wrestling, rugby and football sometimes acquire a condition caused by HSV-1 known as gladiatorial herpes that presents itself as ulceration of the skin of the face, ears and neck. Symptoms include fever, headache, sore throat and swollen glands. It affects the eyes or eyelids from time to time.	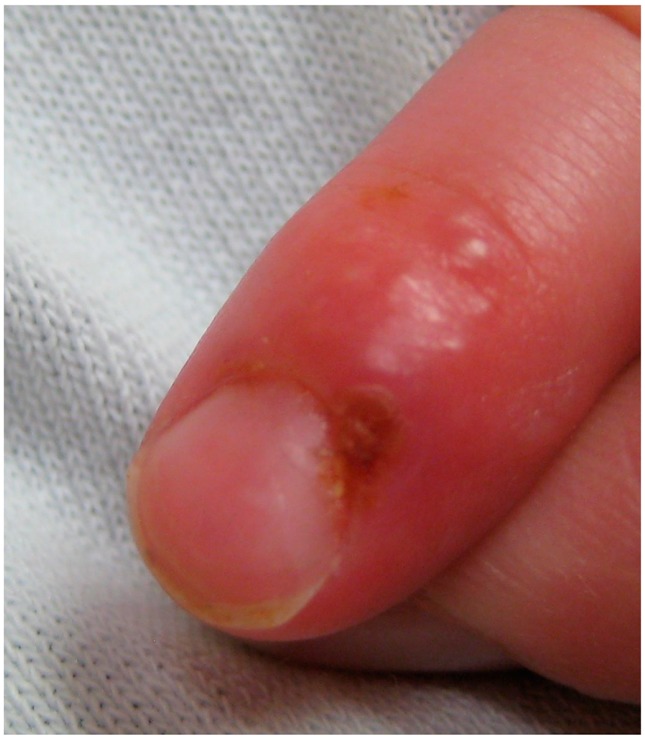 4. Herpetic paterecleris, licence CC BY SA 3.0, adapted for gentle concession of James Heilman, MD.
Herpetic encephalitis or meningitis	Herpetic brain infection is thought to be due to the transmission of viruses from a peripheral site and the following reactivation of HSV-1, along the axon of the trigeminal nerve, to the brain. HSV is the most common cause of viral encephalitis. When the brain is infected, the virus shows a preference for the temporal lobe. HSV-2 is the most common cause of Mollaret’s meningitis, a type of recurrent viral meningitis.	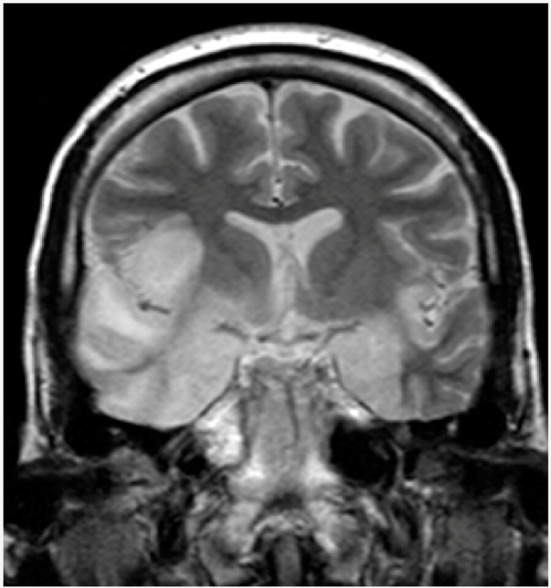 5. Herpes simplex encephalitis, licence CC BY 3.0, adapted with concession of Dr Laughlin Dawes—http://www.radpod.org/2007/03/24/herpes-simplex-encephalitis/.
Herpetic esophagytis	Symptoms may include pain when swallowing (odynophagia) and difficulty swallowing (dysphagia). It is often associated with impaired immune function.	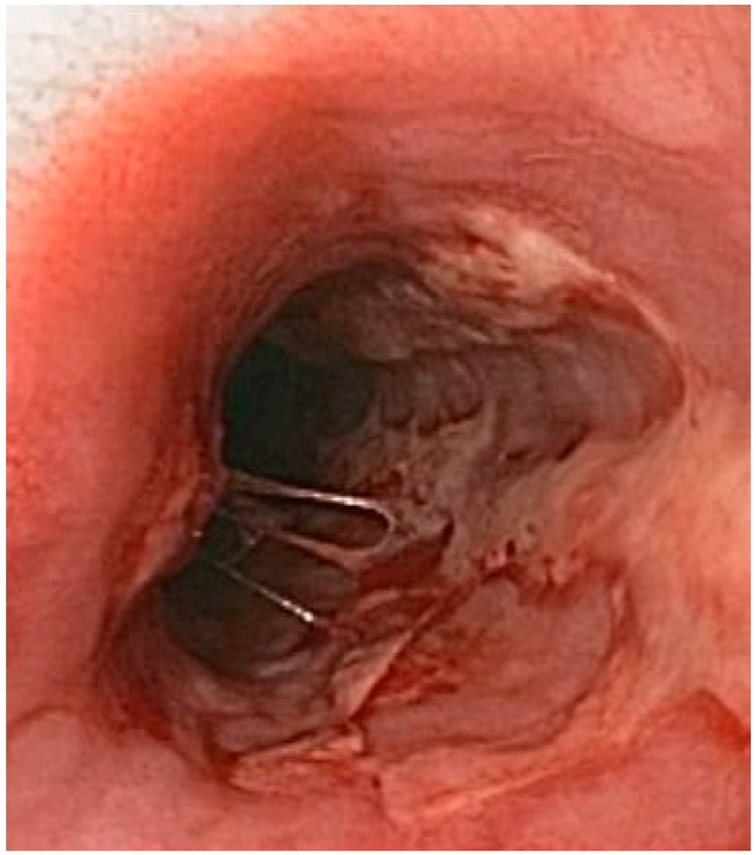 6. Herpetic esophagitis, licence CC BY-SA 3.0, adapted with concession of Donald E. Mansell, MD.

**Table 2:** Randomized clinical trials (RCTs) table with sample groups description and statistical analysis.

Author (Year)	Sample Size	Sample Groups	Posology	Bias Risk	Statistical Results
Polansky et al. [26] (2018)	68	Gene-Eden-VIR/Novirin	1 to 4 capsules per day over a period of 2 to 36 month		Significant
Semprini et al. [27] (2017)	950 proposed	90% kanuka hoey 5% aciclovir	Kanuka honey with 5% aciclovir cream	Open label, parallel group	Protocol study
Palli et al. [28] (2017)	43	Squaric acid dibutyl ester (SADBE) 2% SADBE 0.5% Placebo		Double blind	*p* = 0.009
Batavia et al. [29] (2016)	816 (HIV infected)	Early Antiretroviral therapy Delayed Antiretroviral therapy	Antiretroviral therapy		*p* < 0.01
Zhao et al. [30] (2015)	40	Placebo Averteax	Lipophilic catechins (AverTeaX, Camellix, LLC, Evans, GA, USA) used 6-8 times daily until reduction.	Double blind	*p* < 0.003 QoL reduction *p* = 0.016
You et al. [31] (2015)	144	Inosine pranobex Acyclovir	Active inosine pranobex, 1 g four times daily, and acyclovir placebo Active acyclovir, 200 mg five times daily, and inosine pranobex placebo	Double blind	Not significative total symptom scores Lower recurrence in Test Group
Miller et al. [32] (2014)	171	Complete blood cell counts, standard blood chemistry panels and urinalysis on the samples			*p* < 0.05
Bieber et al. (2014)	775	Acyclovir Placebo	Acyclovir tablet 50mg	Double blind	Significative
Dougall et al. (2013)	87	Low level laser diode Placebo infrared	1072 nm	Double blind	*p* = 0.01
Senti et al. [33] (2013)	40	2-HPbetaCD Placebo	Polyethylene glycol (PEG) formulation containing 20% hydroxypropyl-beta-cyclodextrin (2-HPbetaCD). The gel was applied to the lips twice daily for 6 months.	Double blind	*p* < 0.003
Khemis et al. [34] (2012)	106	CS20 Acyclovir	CS20 (Acura 24 (r)) protective barrier gel	Assessor blinded	*p* = 0.012
Skulason et al. [35] (2012)	150	Monocaprin + doxycycline Monocaprin Placebo	1-monoglyceride of capric acid + doxycylclin for six days 1-monoglyceride of capric acid for six days	Double blind	Healing time *p* = 0.05 Pain *p* = 0.0114
Munoz et al. [36] (2012)	232	Low level light therapy	670-nm laser irradiation, 40 mW, 1.6 J, 2.04 J/cm(2), 51 mW/cm	Double blind	No statistical analysis was performed because of large sample size.
Busch et al. [37] (2009)	105	1,5 pentanediol Placebo	PD gel or placebo gel twice daily to both lips	Double blind	Recurrence time *p* > 0.05 Pain and clinical efficacy *p* = 0.001
